# Intra-cardiac ultrasound guided approach for catheter ablation of typical right free wall accessory pathways

**DOI:** 10.1186/s12872-020-01494-1

**Published:** 2020-05-06

**Authors:** Matevž Jan, Tine Prolič Kalinšek, Jernej Štublar, Matija Jelenc, Andrej Pernat, David Žižek, Nikola Lakič

**Affiliations:** 1 Cardiovascular Surgery Department, University Medical Centre Ljubljana, Zaloška 7, 1000 Ljubljana, Slovenia; 2 Cardiology Department, University Medical Centre Ljubljana, Zaloška 7, 1000 Ljubljana, Slovenia

**Keywords:** Accessory pathway, Right free wall, Intra-cardiac ultrasound, Catheter ablation

## Abstract

**Background:**

Right free wall accessory pathways (AP) are difficult to treat with catheter ablation as ablation catheter (AC) instability at the tricuspid annulus often precludes successful procedure.

The aim of our study was to test a novel intra-cardiac echocardiography (ICE) guided technique for AC placement. Feasibility and success rates were observed.

**Methods:**

Eight consecutive patients (aged 29 ± 21 years, 4 female) with Wolff-Parkinson-White syndrome and a right free wall AP were included in the study. ICE, three-dimensional (3D) electro-anatomic mapping (EAM) system, and a steerable long sheath were used together with either an irrigated or a non-irrigated tip radio-frequency AC to achieve a “loop” manoeuvre which provided AC tip stability at the ventricular aspect of the tricuspid annulus. X-ray fluoroscopy was not used.

**Results:**

Three patients had an anterior and five had a lateral location of the right free wall AP. Procedures were successful in all patients, without recurrences during the mean follow-up of 397 ± 363 days. Average procedural duration was 90 ± 31 min. On average, 6.6 ± 5.7 ablations were needed. Average time to terminate AP conduction after the start of ablation was 4.8 ± 4.2 s. In five patients (62%) AP conduction was successfully terminated with the first ablation. There were no procedural complications.

**Conclusions:**

The novel ICE-guided approach with concomitant use of the steerable sheath and the 3D EAM system for zero-fluoroscopy mapping and ablation of the right free wall APs proved feasible and resulted in excellent acute and long-term outcomes.

## Background

Typical right free wall accessory pathway (AP) mediated tachycardias are relatively rare and associated with higher procedural failure rates compared to left-sided and septal AP locations [1–5]. Different catheter ablation (CA) methods have been proposed to increase acute and long-term success of right free wall AP ablation. Those methods were mostly modifications of mapping approach, including use of a three-dimensional (3D) electro-anatomic mapping (EAM) system, use of multipolar diagnostic catheter for detailed tricuspid annulus mapping, use of detailed atrial insertion mapping and others [6–8]. Recently, fluoroscopy-guided ablation with catheter tip positioning on the ventricular insertion of the AP, beneath the tricuspid valve, was shown to result in excellent procedural and long-term success [9, 10].

The purpose of our work was to assess feasibility of a novel intra-cardiac echocardiography (ICE) guided technique for CA of the right free wall APs without the use of fluoroscopy.

## Methods

### Study population

In this retrospective analysis we included 8 consecutive patients with typical right free wall accessory pathway which underwent radio-frequency (RF) CA between April 2017 and August 2019. All patients had preexcitation clearly visible in the 12 lead electro-cardiogram (ECG) recording and had history of palpitations and supra-ventricular tachycardia recorded on the ECG recording on at least one occasion. Patients with previous CA were also included. Clinical, laboratory examinations and echocardiography were performed before the procedure.

### General procedural characteristics

Most procedures were performed in conscious sedation, except in paediatric patients, where general anaesthesia was used. Right femoral vein punctures were performed to access the heart. All procedures were performed with the three dimensional (3D) electro-anatomic mapping (EAM) system (NavX™, Abbott, Abbott Park, Illinois, USA) and intra-cardiac echocardiography (AcuNav™, Biosense Webster, Irvine, California, USA) without the use of fluoroscopy.

### Initial mapping with the 3D EAM system

As previously described, a 10-polar diagnostic catheter was inserted through the femoral vein and advanced into the right atrium without any use of fluoroscopy and only with the aid of the 3D EAM system [11–14]. Afterwards, a limited 3D anatomy of the right atrium was obtained, position of the His bundle was tagged on the map and finally the catheter was advanced into the coronary sinus. An RF ablation catheter (D curve FlexAbility™, Abbott, Abbott Park, Illinois, USA or D curve Celsius® Biosense Webster, Irvine, California, USA) was advanced into the right atrium through a steerable sheath (large curve Agilis™, Abbott, Abbott Park, Illinois, USA) in a similar manner as the diagnostic catheter. Ventriculo-atrial and atrio-ventricular AP conduction was tested before the start of mapping. Mapping was performed in sinus rhythm or during atrial pacing, depending on which resulted in more preexcitation of the ventricles. A detailed local anatomical and activation map at the earliest ventricular activation at the tricuspid annulus was created. When atrio-ventricular reentry tachycardia (AVRT) was induced and sustained the tricuspid annulus was mapped for the earliest atrial activation.

### Use of the steerable long sheath and ICE for catheter positioning - the “loop” manoeuvre

Once the initial activation mapping revealed the free wall location of the accessory pathway a stable position of the ablation catheter tip was obtained with the following manoeuvre:

- ICE probe was advanced into the right atrium in a fluoroless manner, using the ICE image for guidance.
- Under ICE guidance the steerable long sheath was bent in the septal direction in the case of lateral location of the accessory pathway or in the direction of the posterior tricuspid annulus in the case of anterior location of the accessory pathway.
- Under ICE guidance the ablation catheter was advanced and bent in the direction of the location of the accessory pathway, with its curvature being the opposite of the curvature of the long sheath, with both together forming an open S shape loop.
- Under ICE guidance, slight counterclockwise long sheath rotation and with precise increase or decrease of the curvature of both the steerable sheath and the ablation catheter the tip of the ablation catheter was positioned just beneath the tricuspid annulus.

**Fig. 1:**
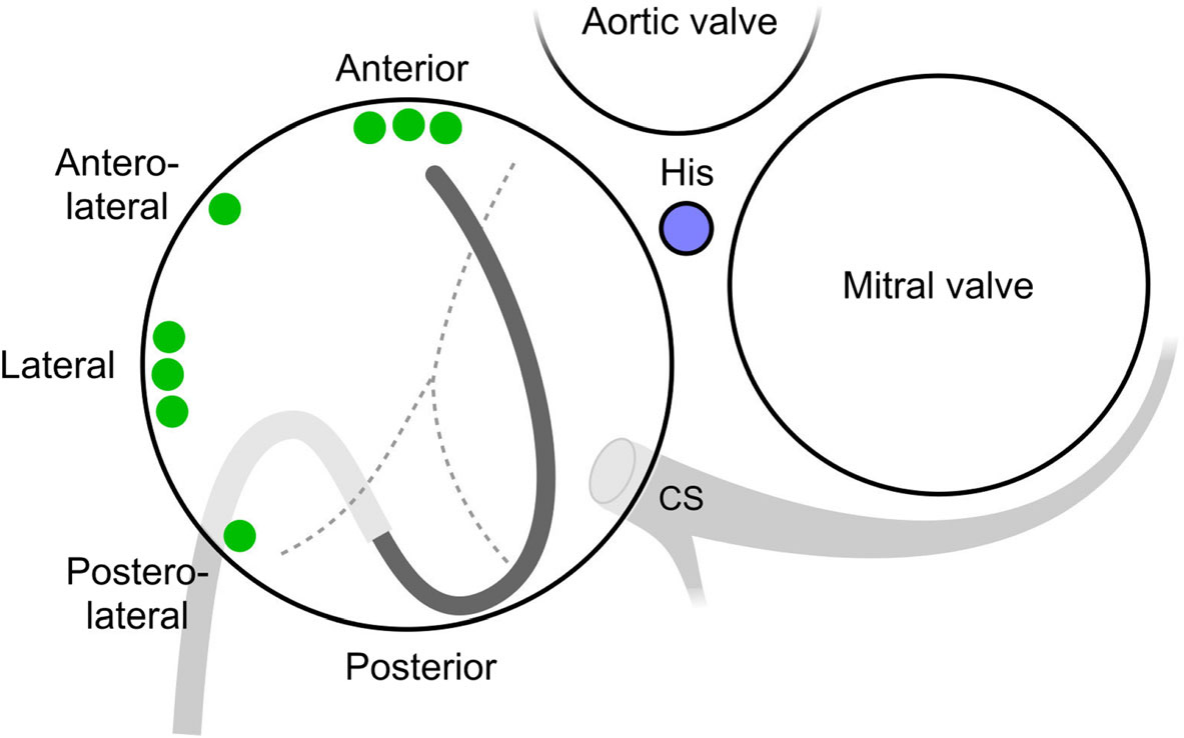
Schematic representation of the “loop” manoeuvre - the steerable long sheath (light grey curve pointing downwards) and the ablation catheter (dark grey curve pointing upwards) position in a case of anterior right free wall accessory pathway ablation. Also, division of the free wall aspect of the tricuspid annulus is shown with green dots representing each individual accessory pathway location. CS coronary sinus.

A schematic example of the “loop” manoeuvre in the case of an anterior right free wall accessory pathway is shown in Fig. 1.

### Detailed mapping and ablation

After a stable position of the ablation catheter tip was obtained some additional mapping points for accurate accessory pathway location were obtained and were typically located slightly toward the ventricular side of the map compared to the mapping points obtained during initial mapping. Finally, RF ablation was performed with 40 W when a non-irrigated tip ablation catheter was used and with 30 W when an irrigated tip ablation catheter was used, both in temperature controlled mode, with cutoff set to 60 and 43 °C, respectively. Ventriculo-atrial and atrio-ventricular AP conduction was tested after successful ablation.

### Procedural parameters

Procedural time was defined as time from the insertion of the venous sheaths to their removal at the end of the ablation procedure. Ablation time was defined as the time spent delivering the RF energy to the myocardium and was automatically recorded by the electrophysiology (EP) recording system. Time needed to achieve accessory pathway conduction block after the start of RF energy delivery was recorded. Number of all ablations per procedure and type of ablation catheter used (irrigated tip versus non-irrigated tip) were recorded. Voltage amplitude of atrial and ventricular endocardial signals at the successful ablation site were also recorded.

### Procedural success and adverse events

Procedural endpoint was defined as anterograde and retrograde block of accessory pathway conduction. All patients had 12 lead ECG recordings immediately after the procedure, the next day, 1 month after hospital discharge and at the scheduled outpatient clinic follow up approximately 6 months after the procedure. Additional 12 lead ECG recordings and 24 h Holter ECG recordings were obtained according to the discretion of the physician performing the follow up.

Peri-procedural adverse events resulting in prolonged or repeat hospitalisation, those requiring surgical intervention or resulting in patient’s long-term disability, were monitored within 30-days from the ablation procedure.

## Results

The study population included 4 males (50%) and 4 females, aged 29 ± 21 years. Detailed baseline patient characteristics are shown in Table 1. 3D EAM system, ICE and long steerable sheath were used in all procedures. X-ray fluoroscopy was not used. In all cases bidirectional AP conduction was present.

**Table 1:** Baseline patient characteristics

Patient number	age range (years)	BMI	Previous procedures (number)
1	10–19	22,0	0
2	10–19	17,3	0
3	40–49	27,1	0
4	10–19	19,0	0
5	30–39	23,1	0
6	20–29	23,9	0
7	70–79	32,7	1
8	10–19	19,5	0

*BMI* body mass index

**Fig. 2:**
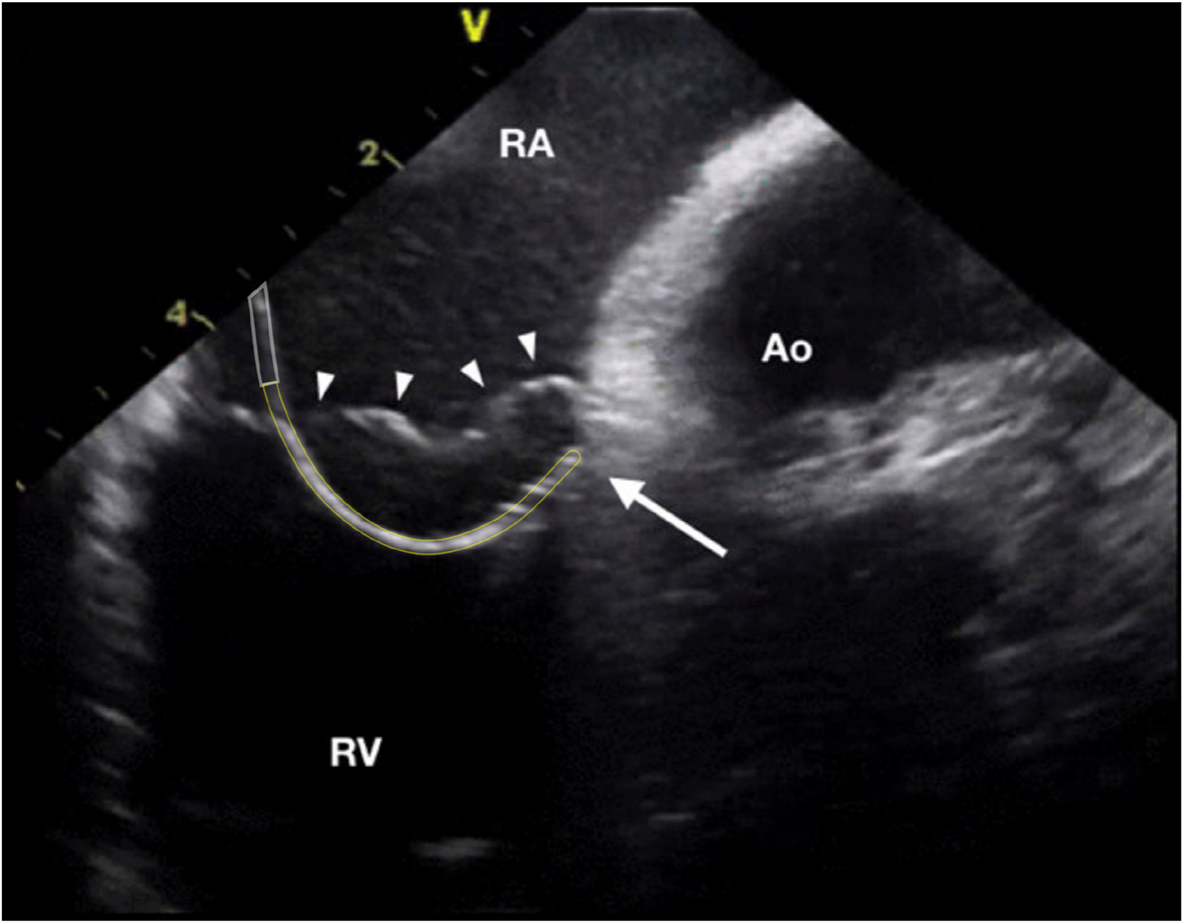
Intra-cardiac echocardiography (ICE) image taken during mapping and ablation of the an-terior right free wall accessory pathway (AP). The 10 Fr (French scale) ICE probe was in-serted into the right atrium via the femoral vein. The ablation catheter was inserted into the right ventricle via a steerable long sheath and the described “loop” manoeuvre was used. White lines mark the presumed location of the steerable long sheath, yellow lines mark the course of the ablation catheter loop. The tip of the ablation catheter is marked with a solid white arrow, the tricuspid valve is marked with solid white arrowheads. RA right atrium, RV right ventricle, Ao aorta.

**Fig. 3:**
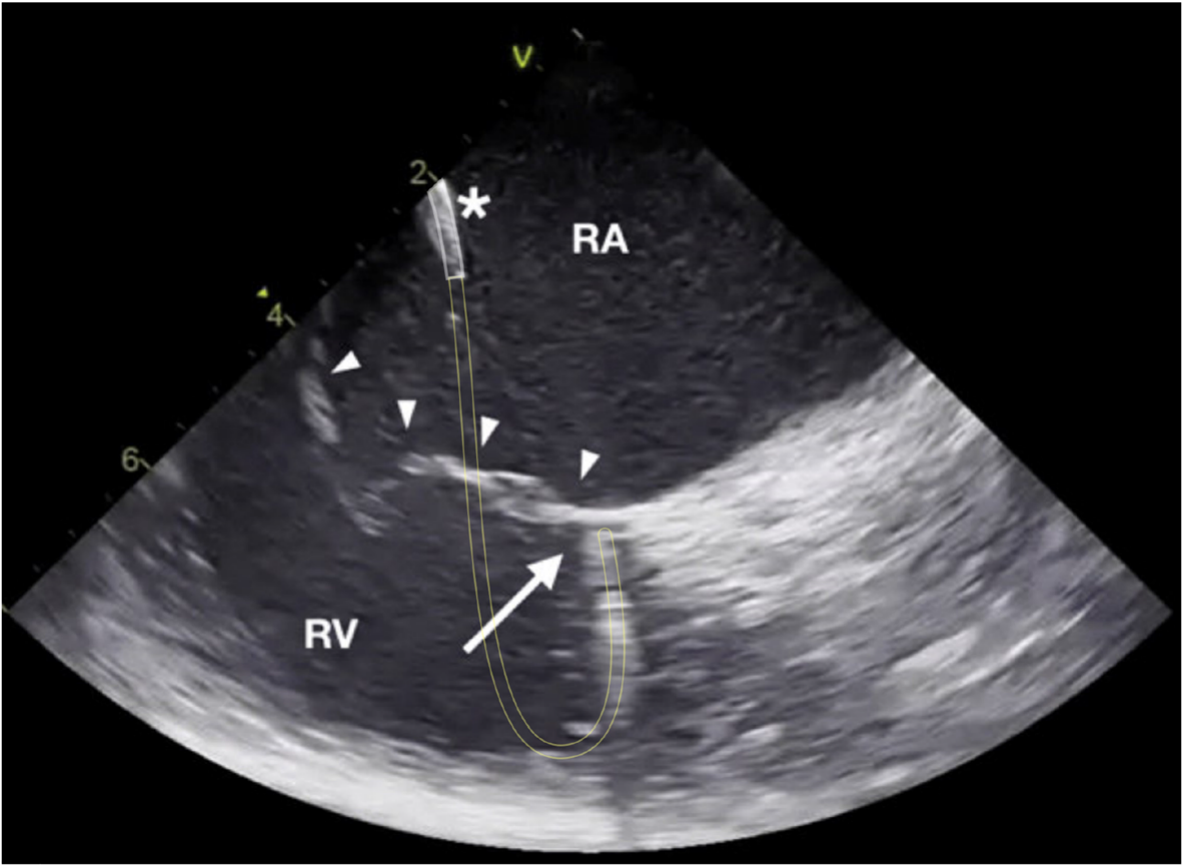
Intra-cardiac echocardiography (ICE) image taken during mapping and ablation of the anter-olateral right free wall accessory pathway (AP). The “loop” manoeuvre was done as de-scribed in Fig. 2. White lines mark the location of the steerable long sheath that is also marked with an asterisk, yellow lines mark the presumed course of the ablation catheter loop. The tip of the ablation catheter is marked with a solid white arrow, the tricuspid valve is marked with solid white arrowheads. RA right atrium, RV right ventricle.

**Fig. 4:**
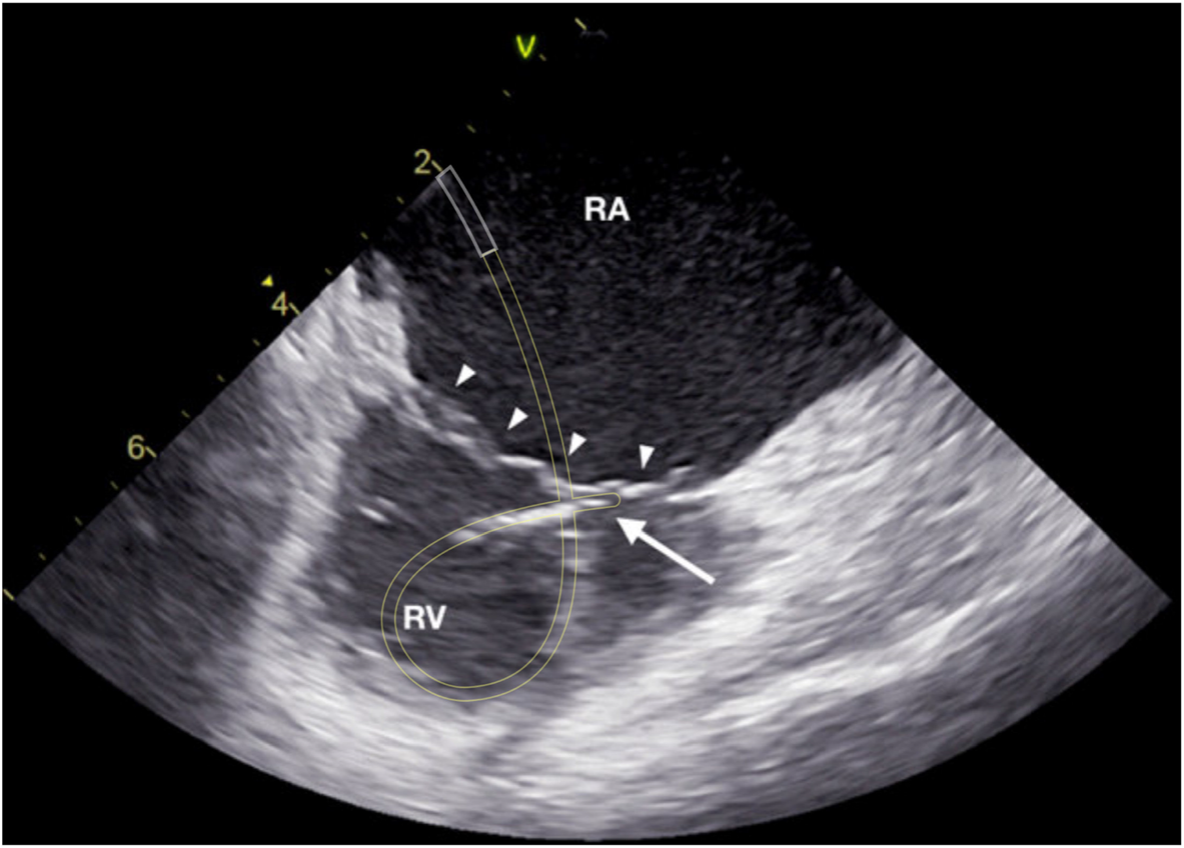
Intra-cardiac echocardiography (ICE) image taken during mapping and ablation of the lateral right free wall accessory pathway (AP). The “loop” manoeuvre was done as described in Fig. 2. White lines mark the presumed location of the steerable long sheath, yellow lines mark the presumed course of the ablation catheter loop. The tip of the ablation catheter is marked with a solid white arrow, the tricuspid valve is marked with solid white arrowheads. RA right atrium, RV right ventricle.

**Fig. 5:**
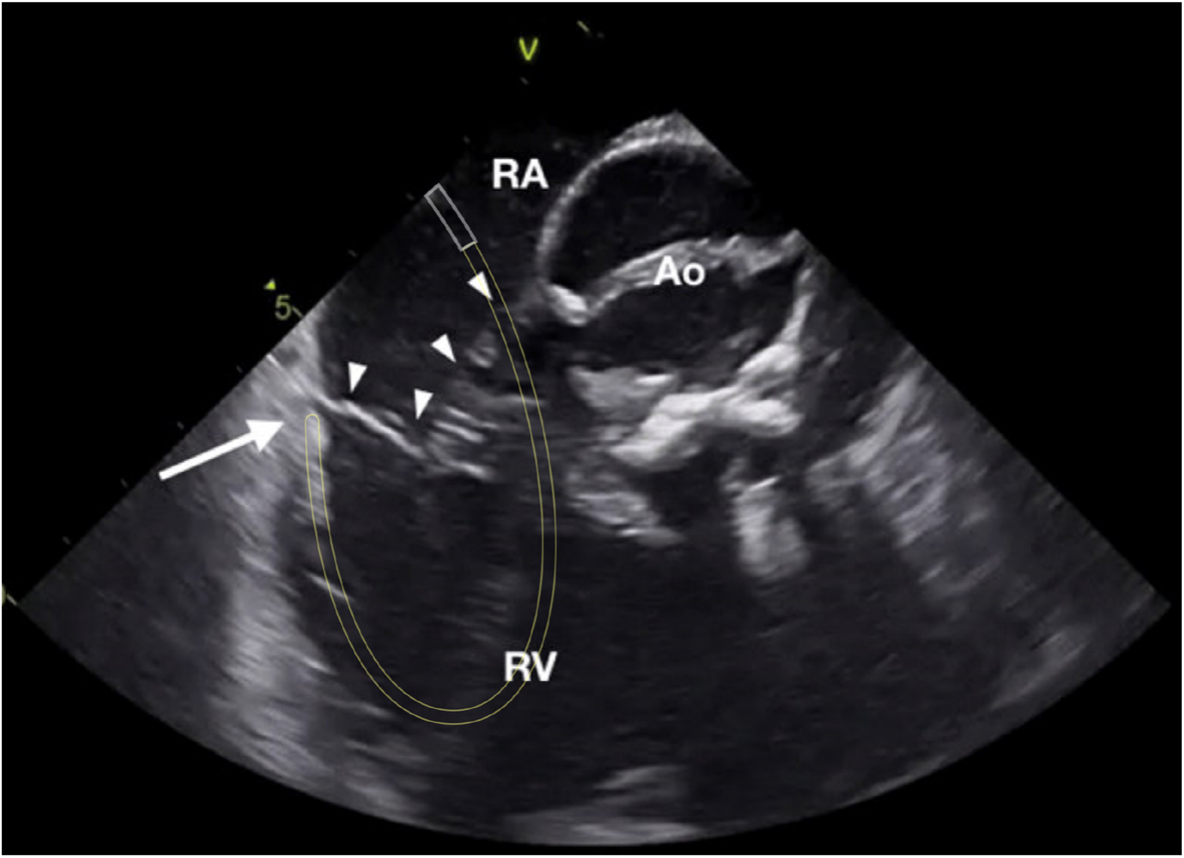
Intra-cardiac echocardiography (ICE) image taken during mapping and ablation of the pos-tero-lateral right free wall accessory pathway (AP). The “loop” manoeuvre was done as de-scribed in Fig. 2. White lines mark the presumed location of the steerable long sheath, yellow lines mark the presumed course of the ablation catheter loop. The tip of the ablation catheter is marked with a solid white arrow, the tricuspid valve is marked with solid white arrowheads. RA right atrium, RV right ventricle, Ao aorta.

Right free wall was arbitrarily divided into four regions depending on the orientation of the loop and the tip of the ablation catheter as seen with the ICE (Fig. 1): 3 patients (37%) had anterior location of the accessory pathway with the loop of the ablation catheter oriented vertically and the tip being lateral from the location of the His bundle (Fig. 2); 1 patient (12%) had an anterolateral location of the accessory pathway with the loop and the tip of the ablation catheter oriented oblique toward the upper right part of the tricuspid annulus (Fig. 3); 3 patients (37%) had a lateral location of the accessory pathway with the loop and the tip being oriented horizontally (Fig. 4); 1 patient (12%) had a postero-lateral location of the accessory pathway with the loop and the tip of the ablation catheter oriented oblique toward the lower right part of the tricuspid annulus (Fig. 5).

One patient with the anterior AP had a failed cryoablation attempt during the same procedure. One patient with postero-lateral AP had a previous RF ablation procedure.

**Fig. 6:**
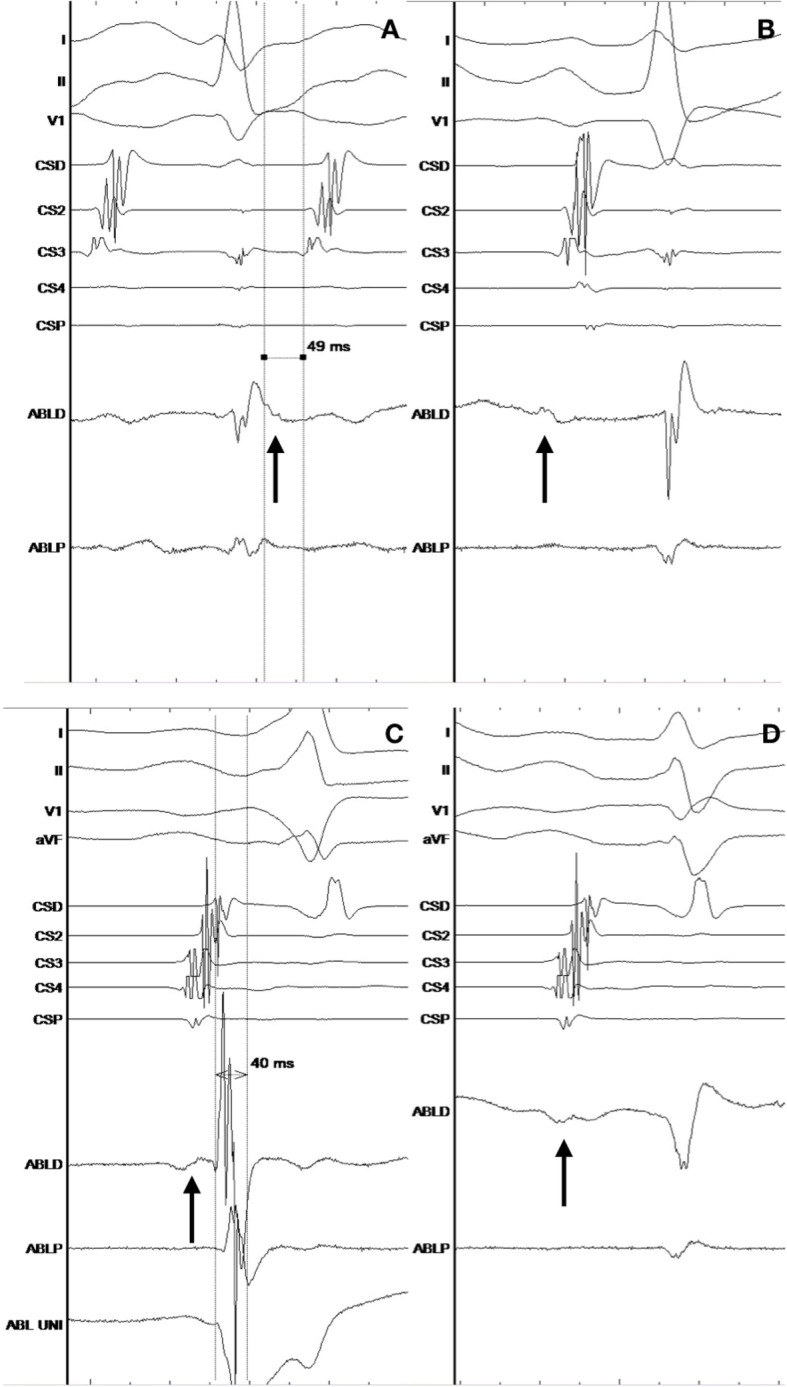
Endocardial electrocardiograms during mapping and ablation at the ventricular aspect of the tricuspid valve annulus. Arrow marks atrial electrocardiogram that has low amplitude and a far-field appearance, ABLD signals from distal tip of the ablation catheter, CSP-CSD signals from 10 polar diagnostic catheter inserted into the coronary sinus. Panel **a** shows signals during mapping of the orthodromic AVRT with an atrial electrocar-diogram that is 49 ms early compared to the reference in the CS3. Panel **b** shows signals during sinus rhythm after ablation at the previous site and termina-tion of the orthodromic AVRT. Panel **c** shows signals during mapping in sinus rhythm in a patient with clear ventricular preexcitation. The large amplitude ventricular signal is 40 ms early compared to the begin-ning of the delta wave in surface electrocardiogram tracings (I, II, V1 and aVF) and is in line with the beginning of the negative deflection in the unipolar recording from the distal tip of the ablation catheter (ABL UNI). Panel **d** shows signal after ablation at the previous site and termination of ventricular preexcitation.

Ventricular insertion of the accessory pathway was mapped during sinus rhythm in 7 (87%) patients (Fig. 6, Panels C and D). Only in one patient mapping was performed during ongoing orthodromic AVRT, with annotation of the earliest far-field atrial electrogram recorded at the ventricular side of the tricuspid annulus (Fig. 6, Panels A and B).

Average procedural time was 90 ± 31 min. On average 6.6 ± 5.7 ablations were needed with an average ablation time of 236 ± 109 s. In 5 patients (62%) the first RF ablation was already successful in blocking the AP conduction. Average time to AP conduction block after start of the successful RF application was 4.8 ± 4.2 s. Average endocardial atrial signal amplitude at the successful ablation site was 0.16 ± 0.13 mV with an average atrial to ventricular voltage amplitude ratio of 0.15. Detailed procedural parameters are shown in Table 2.

**Table 2:** Procedural parameters

Patient number	Procedural duration (min)	AP location	I/NI RF or Cryo	TT (s)	Mapping in SR or AVRT	Number of RF lesions	Number of RF lesions before AP block	RF ablation time (s)	Atrial signal amplitude at successful ablation site (mV)	Atrial to ventricular signal ratio at successful ablation site
1	120	A	NI	10	SR	20	17	447	0.30	0.3
2	80	A	NI	2	SR	3	0	140	0.37	0.3
3	115	L	NI	1	SR	9	0	283	0.05	0.01
4	65	AL	I	4	AVRT	4	0	203	0.08	0.08
5	145	A	Cryo failed, I	2	SR	4	0	145	0.15	0.06
6	60	L	I	4	SR	4	2	193	0.09	0.17
7	65	PL	I	3	SR	3	0	146	0.06	0.17
8	75	L	NI	13	SR	6	1	331	0.10	0.1

*AP* accessory pathway, *I/NI* irrigated or non-irrigated tip ablation catheter, *RF* radio-frequency, Cryo cryoablation, *TT* time to termination of AP conduction after start of ablation, *SR* sinus rhythm, *AVRT* atrio-ventricular reentry tachycardia

Acute endpoint was reached in all procedures and there were no complications. During the average follow-up of 397 ± 363 days all patients (100%) were free of preexcitation and had no clinical tachycardias.

## Discussion

Our novel ICE-guided technique for CA of the right free wall APs showed promising acute and long-term results. In addition, there were no peri-procedural adverse events.

In general, catheter instability is the major reason for unsuccessful CA of the right free wall accessory pathways. Catheter instability at the tricuspid annulus produces difficulties in mapping the atrial or ventricular insertion of the accessory pathway and failure to apply adequate RF current [15]. To some extent, when using transfemoral approach, catheter instability can be overcome with mapping the atrial insertion during ongoing orthodromic AVRT or ventricular pacing using the 3D EAM or a linear multipolar catheter positioned around the tricuspid annulus [6–8]. According to recent reports, the fluoroscopy-guided transjugular approach can help with stabilization of the ablation catheter tip in case of right anteroseptal, anterior and anterolateral accessory pathways, resulting in high procedural success rates and no recurrences [16, 17]. In our study we introduce a novel “loop” manoeuvre, which enabled transfemoral mapping of the ventricular aspect of the tricuspid annulus, which was mainly performed during sinus rhythm (ante-grade activation of the accessory pathway). In one patient we were able to map the atrial activation at the ventricular side of the annulus during ongoing orthodromic AVRT. However, atrial activation mapping from the ventricular side of the annulus can be less accurate due to the low amplitude of far-field atrial signals. Clearly, the near-field and large amplitude ventricular signals are more appealing to map and are the preferred target when mapping the ventricular aspect of the tricuspid valve annulus.

Steerable long sheath and ablation catheter placement was performed mainly under ICE control, which enabled accurate positioning of the ablation catheter tip to the target area beneath the tricuspid valve. This approach showed to be very useful in a zero-fluoroscopy setting since relying mostly on 3D EAM system and endocardial electrograms could prove misleading due to possible low voltage atrial signals at the ventricular aspect of the annulus. However, 3D EAM system seems to be an indispensable mapping tool, since ICE enables the operator to visualise detailed anatomy, but cannot show overall anatomy and activation pattern during catheter ablation of the right free wall APs.

Although the sub-annular region of the tricuspid valve is regarded as a possible low flow area, the use of non-irrigated RF ablation catheter proved satisfactory. There were no issues with temperature surge and power delivery in the four procedures that were performed with the non-irrigated ablation catheter.

Similar fluoroscopy-guided technique of sub-valvular mapping and ablation was recently described [9, 10]. Both studies included a small number of patients with right free wall AP. In the study by Wieczorek et al. there were 7 patients with right free wall AP. Steerable long sheath was only used when deemed necessary and AP block was achieved in approximately 6 s of RF ablation with average procedural time of 63 min and 18 min of fluoroscopy. In the second study by Yang et al., the 3D EAM system was used in only one of 7 patients with right free wall AP. They were able to reach AP block on average in approximately 6 s of RF ablation. Procedural time was 86 min and fluoroscopy time was 9 min. Compared to both studies we were able to reach slightly shorter RF ablation times, which could be the result of ICE-guided visual control of the catheter tip orientation, location and contact with the sub-valvular region at the ventricular aspect of the tricuspid valve. On the other hand, slightly longer duration of procedures in our study could be the result of more time-consuming catheter positioning using the 3D EAM and ICE.

There are some obvious limitations of our study. First, a small number of patients limits the value of our conclusions. Second, mapping and ablation was mostly targeting the ventricular insertion of the right free wall APs and our findings cannot be translated to atrial insertion mapping and ablation. Third, mapping was mostly performed during sinus rhythm and in patients with bidirectional AP conduction. Finally, our mapping approach might not be adequate in patients with only retrograde AP conduction.

## Conclusions

The novel ICE-guided approach with concomitant use of the steerable sheath and the 3D EAM system for zero-fluoroscopy mapping and ablation of the right free wall APs proved feasible and resulted in excellent acute and long-term outcomes.

## Data Availability

Data generated or analysed during this study is for the most part included in this published article. Additional raw data is available and can be shared upon request to the corresponding author.

## Abbreviations

AP: Accessory pathway
AC: Ablation catheter
3D: Three dimensional
EAM: Electro-anatomic mapping
ICE: Intra-cardiac echocardiography
ECG: Electro-cardiogram
AVRT: Atrio-ventricular reentry tachycardia
RF: Radio-frequency
EP: Electrophysiology
